# Association of serum chemokine ligand 21 levels with asthma control in adults

**DOI:** 10.6061/clinics/2021/e1713

**Published:** 2021-01-11

**Authors:** Yueyan Lou, Yu Zheng, Yanhua Xu, Hui Lu, Yiwei Wang, Yinshi Guo

**Affiliations:** I Department of Allergy, Renji Hospital, School of Medicine, Shanghai Jiao Tong University, Shanghai 200127, China; II Department of Pulmonology, Renji Hospital South Campus, School of Medicine, Shanghai Jiao Tong University, Shanghai 200127, China

**Keywords:** Chemokine ligand (CCL) 21, Inflammatory Cytokines, Asthma

## Abstract

**OBJECTIVES:**

The chemokine ligand (CCL) 21 regulates the maturation, migration, and function of dendritic cells, and has been implicated in the pathogenesis of asthma. This study aimed to investigate the association between serum CCL21 levels and asthma control.

**METHODS:**

The serum levels of CCL21 and other inflammatory cytokines were analyzed in patients with asthma (n=44) and healthy controls (n=35) by enzyme-linked immunosorbent assay. IgE levels and eosinophil counts were determined by turbidimetric inhibition immunoassay and fully automatic blood analysis, respectively. The Asthma Control Test (ACT) questionnaire was used, and spirometry and fractional exhaled nitric oxide (FENO) measurements were performed. A multiple unpaired Student’s *t*-test was performed to analyze the differences in CCL21 and interleukin levels between patients with asthma and healthy controls. The correlation of CCL21 levels with disease severity was evaluated using the Pearson’s rank correlation test.

**RESULTS:**

Serum CCL21 levels were lower in patients with asthma (254.78±95.66 pg/mL) than in healthy controls (382.95±87.77 pg/mL) (*p*<0.001). Patients with asthma had significantly higher levels of IL-1β (19.74±16.77 *vs.* 2.63±5.22 pg/mL), IL-6 (7.55±8.65 *vs.* 2.37±2.47 pg/mL), and tumor necrosis factor-α (12.70±12.03 *vs.* 4.82±3.97 pg/mL) compared with the controls. CCL21 levels were positively correlated with the ACT score (r_s_=0.1653, *p*=0.0062), forced expiratory volume in 1s (FEV1)/forced vital capacity (r_s_=0.3607, *p*<0.0001), and FEV1 (r_s_=0.2753, *p*=0.0003), and negatively correlated with FENO (r_s_=0.1060, *p*=0.0310). CCL21 levels were negatively correlated with serum IgE levels (r_s_=0.1114, *p*=0.0268) and eosinophil counts (r_s_=0.3476, *p*<0.0001).

**CONCLUSIONS:**

Serum CCL21 levels may be a new biomarker for assessing asthma control.

## INTRODUCTION

Asthma can cause reversible airway obstruction and airway hyperresponsiveness. It is a disease characterized by an inflammatory response in the airway. In allergic asthma, the immune response induced by inhaled antigens tends to be the T helper 2 response (1,2). Dendritic cells (DCs) work as professional antigen-presenting cells. The antigen-presenting process of DCs induces allergic inflammation (3,4). Immature DCs possess the ability to take up and process antigens, but lack the ability to present antigens (5). Immature DCs mature after antigen presentation and can express CD86, CD80, CD40, and chemokine receptors (CCRs) (6). By interacting with CCR7 and chemokine ligand 21 (CCL21, also known as secondary lymphoid chemokine), mature DCs can migrate to lymph nodes and effectively present antigens to naive T cells. CCL21 plays an important role in the aggregation and induction of naive T cells, B cells, and DCs (7,8). CCL21 is expressed in high endothelial venules around the bronchus and lymphatics around the pulmonary vessels.

IL-1β is the main secretory subtype of IL-1, which can mediate the development of allergic diseases and asthma (9) via differentiation and activation of Th2 cells (10) and Th17 cells. It has been shown that patients with asymptomatic asthma may have higher blood IL-6 levels (11). Neutrophils, activated lymphocytes, natural killer cells, endothelial cells, mast cells, and mononuclear phagocytes all produce tumor necrosis factor (TNF)-α, which is involved in the pathogenesis of asthma. TNF-α levels in patients with severe asthma are higher than those in patients with mild to moderate asthma and controls (12). In this study, we examined the serum levels of CCL21, IL-1β, IL-6, and TNF-α in patients with asthma and healthy controls. We assessed the possibility of using CCL21 as a biomarker to predict disease activity.

## METHODS

### Patients and controls

This is a cross-sectional case-control study carried out between May 2017 and July 2018. The study included 44 patients with asthma (Group I) and 35 age- and sex-matched healthy controls (Group II). Patients with tuberculosis or any other associated respiratory/systemic diseases, and patients with more than 10 pack-years were excluded from the study. Patient selection was carried out according to the Global Initiative for Asthma (GINA) guidelines (13). The Institutional Ethics Committee of Renji Hospital approved the study (2014-081k). Oral informed consent was obtained from all participants.

### Measurement of cytokine levels

Serum cytokine levels were determined by enzyme-linked immunosorbent assay (ELISA) (Dakewe Biotech Co., Ltd, Shenzhen, China). The human Th1/Th2/Th17 cytokine kit was used to quantify the serum concentrations of IL-1β, IL-6, and TNF-α. Serum CCL21 in the supernatant was quantified using a human CCL21 ELISA kit (Abcam, UK).

### Pulmonary function tests

In accordance with the ATS/ERS standard, we measured the pulmonary function of patients with asthma using a Jaeger system (Germany) (14). The predicted forced expiratory volume in 1s (FEV1) and the ratio of FEV1 to forced vital capacity (FEV1/FVC) were derived from the best of three repeatable forced expirations.

### Exhaled nitric oxide measurements

The fractional exhaled nitric oxide (FENO) was analyzed using a NIOX Vero (UK) instrument following the recommendations of the ATS/ERS (14). A FENO cutoff value of 25 ppb was adopted, as recommended by ATS.

### Measurement of IgE levels and eosinophil counts

Serum immunoglobulin E (IgE) levels were measured using a turbidimetric inhibition immunoassay. Eosinophil counts were determined using a fully automatic blood analyzer.

### Statistical analysis

The multiple unpaired Student’s *t*-test was used to compare serum cytokine levels between patients and healthy controls. Serum CCL21 levels were compared between different asthma control groups using one-way analysis of variance (ANOVA). The relationships between cytokine levels were investigated using Pearson’s correlation coefficient test. Correlations between serum cytokine levels and clinical characteristics were analyzed using Pearson’s correlation coefficient test. All data were analyzed using SPSS 23.0 software or GraphPad 6 software. A two-tailed *p*-value of <0.05 was regarded as statistically significant in all analyses.

## RESULTS

### Clinical characteristics of the study subjects

In total, 44 patients and 35 healthy controls were enrolled. Demographics and clinical characteristics are shown in Table 1. There was no significant difference between the two groups in terms of sex and age composition (*p*>0.05). The five-item Asthma Control Test (ACT) questionnaire was used to measure asthma control (15). Each item contained five answer options. In accordance with the GINA 2018 guidelines (16), ACT scores of 5-15 were considered as poorly controlled asthma, scores of 16-19 were considered as not well-controlled asthma, and scores of 20-25 were considered to be well-controlled asthma (Table 1). Lung function parameters, FENO, and serum indices are also listed in Table 1. The medication use of patients with asthma is also shown in Table 1.

### Serum levels of CCL21 in asthma and control groups

**Figure 1:**
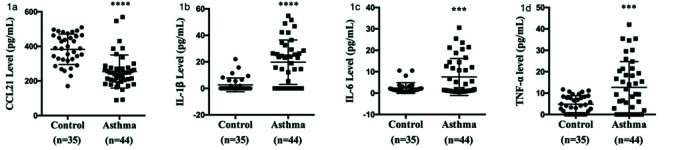
Comparison of serum cytokine levels between patients with asthma and healthy controls. (a) Serum CCL21 levels were determined by ELISA. (b) IL-1β, (c) IL-6, and (d) TNF-α levels were analyzed by cytometric beads array. Statistical analysis was performed using the multiple unpaired Student’s *t*-test. ***p<0.01, ****p<0.001. CCL, chemokine ligand; ELISA, enzyme-linked immunosorbent assay; TNF-α, tumor necrosis factor-α.

**Figure 2:**
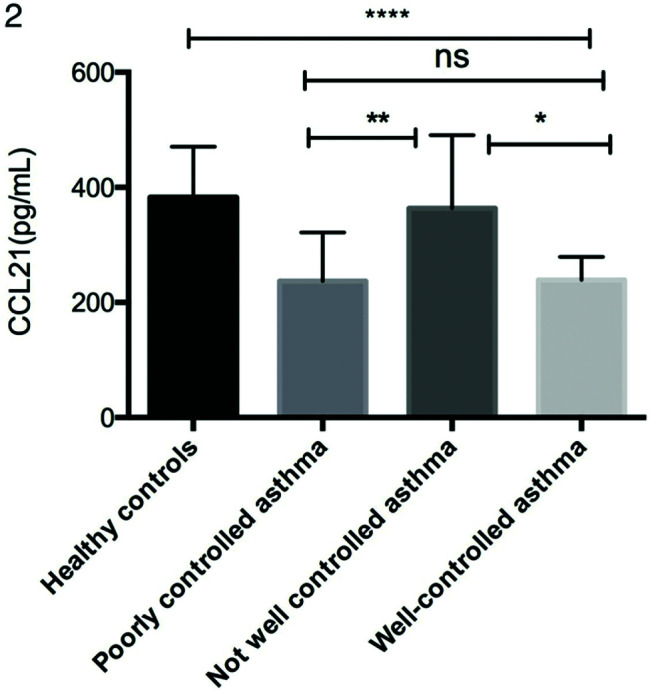
Comparison of serum CCL21 levels in different asthma control subgroups. Serum CCL21 levels were determined by ELISA. Statistical analysis was performed by one-way ANOVA. *p<0.05, **p<0.01, ****p<0.0001. ANOVA, analysis of variance; ELISA, enzyme-linked immunosorbent assay; CCL, chemokine ligand.

As shown in Figure 1a, serum CCL21 levels were lower in patients with asthma (254.78±95.66 pg/mL) than in the healthy controls (382.95±87.77 pg/mL). Moreover, the CCL21 levels were significantly different between the not well-controlled asthma, poorly controlled asthma, and well-controlled asthma subgroups (Figure 2). As shown in Figure 1b-d, patients with asthma had significantly higher levels of IL-1β (19.74±16.77 *vs.* 2.63±5.22 pg/mL), IL-6 (7.55±8.65 *vs.* 2.37±2.47 pg/mL), and TNF-α (12.70±12.03 *vs.* 4.82±3.97 pg/mL) than the controls.

### Correlation between serum CCL21 levels and the clinical parameters of patients

**Figure 3:**
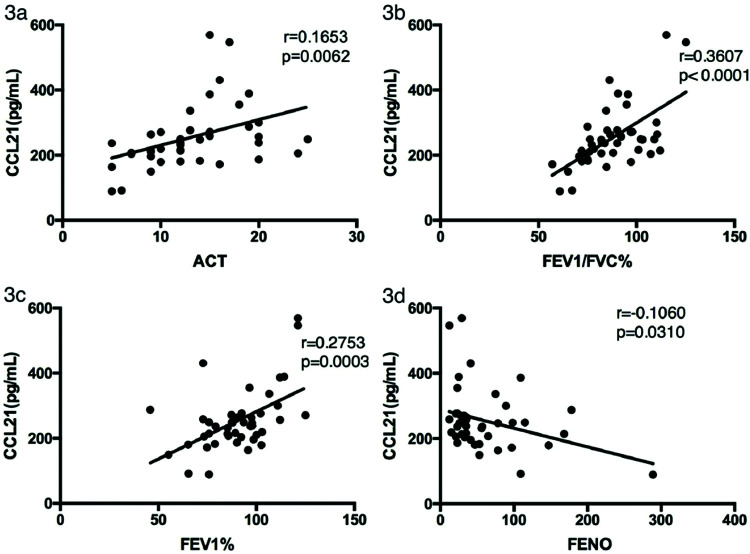
Correlation between CCL21 levels and clinical characteristics in patients with asthma. The correlation analysis was performed to analyze the relationship between CCL21 levels and (a) ACT score, (b) FEV1, (c) FEV1/FVC%, and (d) FENO. ACT, Asthma Control Test; FEV1, forced expiratory volume in 1 second; FVC, forced vital capacity; FENO, fractional exhaled nitric oxide; CCL, chemokine ligand.

We analyzed the relationships between CCL21 levels in asthma patients and their ACT scores, lung function parameters, and FENO. As shown in Figure 3a-d, CCL21 levels were significantly positively correlated with the ACT score (r_s_=0.1653, *p*=0.0062), FEV1/FVC (r_s_=0.3607, *p*<0.0001), and FEV1 (r_s_=0.2753, *p=*0.0003), and negatively correlated with FENO (r_s_=0.1060, *p=*0.0310).

### Correlation between serum CCL21 and IgE levels and eosinophil counts of patients

**Figure 4:**
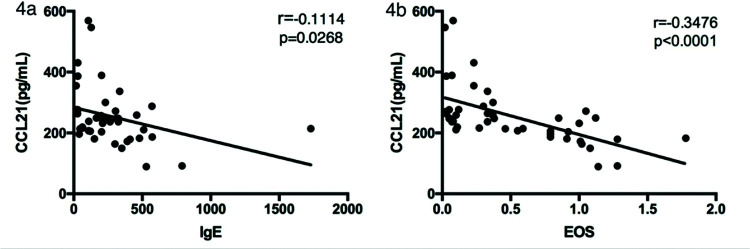
Correlation between serum CCL21 and IgE levels and eosinophil counts in patients with asthma. The correlation analysis was performed to analyze the relationship between CCL21 levels and (a) serum IgE levels and (b) eosinophil counts. CCL, chemokine ligand.

We next analyzed the relationship between serum CCL21 and IgE levels and eosinophil counts in asthma patients. As shown in Figure 4a and b, the CCL21 levels were significantly negatively correlated with serum IgE levels (r_s_=0.1114, *p=*0.0268) and eosinophil counts (r_s_=0.3476, *p*<0.0001).

## DISCUSSION

In this study, for the first time, we found that CCL21 levels in patients with asthma were lower than those in healthy controls, and the levels of IL-1β, IL-6, and TNF-α were higher in patients with asthma than in healthy controls. The role of DCs in the pathogenesis of asthma has been studied, but the effects of CCL21 on DCs and the pathogenesis of asthma are not clear. It has previously been shown that *in vivo* neutralization of CCL21 could prevent human DC migration to the cervical lymph nodes in mucocutaneous lymph node syndrome in humanized severe combined immunodeficient mice and the subsequent development of asthma features (17). It has been suggested that CCL21 is sufficient to mediate DC migration, maturation, and function, and DC has synergistic effects with chemokine receptors and chemokine ligands in the maturation of DCs (17). DCs can present antigens after maturation and release a large number of inflammatory factors, such as TNF-α, IL-1β, and IL-6 (18). Jonuleit et al. (19) found that IL-1β, IL-6, and TNF-α are sufficient to induce DC maturation. Therefore, we measured IL-1β, IL-6, and TNF-α levels to indirectly verify the maturity of DCs in patients with asthma. At the same time, we measured and found lower serum CCL21 levels in patients with asthma than in healthy controls. During maturation, DCs upregulate the surface expression of the chemokine receptor CCR7. Ligands for CCR7, the chemokines CCL19 and CCL21, constitutively expressed at high levels in the lymph nodes are powerful attractants that direct DCs to these tissues (8,20). Because these ligands interact with CCR7, the serum levels of dissociated CCL21 were lower in patients with asthma than in healthy controls. However, the specific relationship between CCL21 and DCs in asthma requires further exploration at the cellular level.

We also analyzed the relationships between CCL21 levels and the ACT score, pulmonary function parameters, and FENO in patients with asthma. The levels of CCL21 were positively correlated with the ACT score (r_s_=0.1653, *p*=0.0062), FEV1/FVC (r_s_=0.3607, *p*<0.0001), and FEV1 (r_s_=0.2753, *p=*0.0003), and negatively correlated with FENO (r_s_=0.1060, *p=*0.0310). CCL21 levels were lower in the poorly controlled and well-controlled asthma groups than in the control group, but no significant difference was found between the poorly controlled group and the well-controlled group. However, we found a positive correlation between CCL21 levels and ACT scores. We speculate that there may be some relationship between and with the grouping criteria. The correlation between serum CCL21 levels and pulmonary function suggests that CCL21 can predict asthma control in patients, but further research is still required.

Finally, we assessed the relationship between serum CCL21 and IgE levels and eosinophil counts in patients with asthma. We found that CCL21 levels were negatively correlated with IgE levels (r_s_=0.1114, *p=*0.0268) and eosinophil counts (r_s_=0.3476, *p*<0.0001). It has been proved that the expression of CCR7 by DCs from the ocular surface is necessary for subsequent IgE production (21). Human eosinophils express CCR7 on their surface, and CCL21 plays a role in chemotaxis in eosinophil migration. The overall eosinophil levels were higher in patients with asthma, and they expressed CCR7 on their surface; all these factors influence serum CCL21 levels in patients with asthma (22). Grinnan et al. found that the induction of allergic asthma in mutant mice with impaired CCR7 response results in characteristics similar to severe asthma in human patients, including severe bronchial lymphocytosis, eosinophilia, and neutrophilia (23). Hence, we found that the serum CCL21 levels in patients with asthma were lower than those in healthy controls, and CCL21 levels were negatively correlated with serum IgE levels and eosinophil counts, which was consistent with previous research.

In conclusion, we found that serum CCL21 levels may serve as a biomarker for asthma control in clinical studies, but further studies of the specific molecular mechanisms are needed to verify this hypothesis.

## AUTHOR CONTRIBUTIONS

All of the authors were involved in drafting the manuscript or critically revising it for important intellectual content, and all of the authors approved the final version to be submitted for publication. Guo Y had full access to all of the data in the study and takes responsibility for the integrity of the data and the accuracy of the data analysis. Lou Y, Zheng Y, Guo Y were responsible for the study conception and design. Xu Y, Lu H and Wang Y were responsible for the data acquisition. Lou Y and Zheng Y were responsible for the analysis and interpretation of data.

## Figures and Tables

**Table 1:** Clinical characteristics of the participants included in the study.

Parameter	Patients with asthma (n=44) Mean±SD	Healthy controls (n=35) Mean±SD
Age, years	38.68±2.47	33.03±1.99
Males, n (%)	26 (59.1%)	22 (62.9%)
Allergic rhinitis, n (%)	23 (52.3%)	/
Smoking history#, n (%)	18 (40.9%)	/
ACT score	13.09±4.950	
Poorly controlled asthma (n=32)	10.69±3.063	/
Not well controlled asthma (n=6)	17.50±1.378	/
Well-controlled asthma (n=6)	21.50±2.345	/
Blood eosinophil counts (×10^9^/L)	0.51±0.46	NOT PERFORMED
IgE levels (IU/mL) (<165)	266.51±292.9	NOT PERFORMED
FEV1/FVC	88.10±15.36	NOT PERFORMED
FEV1	90.67±17.24	NOT PERFORMED
FENO (ppb)	60.25±54.14	NOT PERFORMED
CCL21 (pg/mL)	254.78±95.66	382.95±87.77***
IL-1β (pg/mL)	19.74±16.77	2.63±5.22***
IL-6 (pg/mL)	7.55±8.65	2.37±2.47**
TNF-α (pg/mL)	12.70±12.03	4.82±3.97***
Medication use, n (%)		
Montelukast	8 (18.2%)	/
ICS+LABA(budesonide/formoterol 160/4.5 ug)Twice daily	20 (45.5%)	/
ICS+LABA(budesonide/formoterol 160/4.5 ug)Twice daily+montelukast	16 (36.4%)	/

ACT, Asthma Control Test; FEV1, forced expiratory volume in 1 second; FVC, forced vital capacity; FENO, fractional exhaled nitric oxide; SD, standard deviation.

** Note: *p*<0.01 *vs.* control group

*** *p*<0.001 *vs.* control group.

# patients who smoke less than 10 pack-years.
